# Comparison of the Retention and Fit of Polyether Ether Ketone Clasps during Fatigue Circulation Tests^☆^

**DOI:** 10.1016/j.heliyon.2023.e19959

**Published:** 2023-09-07

**Authors:** Su Wu, Chao Qian, Ting Jiao, Jian Sun

**Affiliations:** Department of Prosthodontics, Shanghai Ninth People's Hospital, Shanghai Jiao Tong University School of Medicine; College of Stomatology, Shanghai Jiao Tong University, National Center of Stomatology, National Clinical Research Center for Oral Diseases, Shanghai Key Laboratory of Stomatology, Shanghai Research Institute of Stomatology, Shanghai, PR China

**Keywords:** Polyether ether ketone (PEEK), Clasp, Retention, Fatigue, Fit

## Abstract

**Purpose:**

The purpose of this study was to evaluate the fit and retention of clasps made of polyether ether ketone (PEEK) or cobalt–chromium alloy (Co–Cr) at different tooth positions in experimental simulations of in vitro wear and removal for 5 years.

**Methods:**

Standard crowns of the right mandibular first premolar (44) and first molar (46) were selected, and a circular three-arm clasp was designed, scanned and fabricated. Ten PEEK clasps were used as the experimental group, and 10 Co–Cr clasps were used as the control group. The seating channel was parallel to the side of the abutment base in both groups. The oral environment was simulated, and each clasp was tested in artificial saliva for 7200 cycles while the change in clasp retention force was recorded. The fit before and after the fatigue cycles was measured by the silicone rubber film copying method. Data were statistically analyzed using the independent samples *t*-test (α = 0.05).

**Results:**

Before circulation, the retention forces of the clasps at position 44 were 4.61 ± 0.91 N (PEEK) and 47.50 ± 10.59 N (Co–Cr), and the forces at position 46 were 3.38 ± 0.49 N (PEEK) and 28.79 ± 10.99 N (Co–Cr). After circulation, the retention forces of the clasps at position 44 were 4.15 ± 0.91 N (PEEK) and 13.90 ± 6.59 N (Co–Cr), and the forces at position 46 were 2.93 ± 0.25 N (PEEK) and 11.56 ± 3.93 N (Co–Cr). Before circulation, the fit of each clasp at the reference points (clasp tip, clasp arm, and occlusal rest) was between 41.70 μm and 170.29 μm, and after circulation, they were between 64.05 μm and 182.59 μm. The retention force and fit of the PEEK clasps did not undergo statistically significant changes from before to after circulation (P > 0.05). However, there were statistically significant (P < 0.05) decreases in the retention force of the Co–Cr clasps and the fit of the clasp tip during circulation. In addition, there was a sudden and large change in the retention force of the Co–Cr clasps after approximately 360 cycles.

**Conclusions:**

The retention force and suitability of the PEEK clasps met the requirements for clinical use during testing that simulated the in vitro wear and removal procedure for 5 years. Compared with the Co–Cr clasp, the PEEK clasp underwent less fatigue deformation, which makes it feasible for clinical applications.

## 1 Introduction

At present, the removable partial denture (RPD) used in clinical applications is typically composed of a plastic base combined with a metal stent and clasp [1]. However, this design often cannot account for both function and aesthetics [2]. At present, clinical dental patients are interested in more than the recovery of function [3]. They are often dissatisfied with the aesthetics of denture restorations due to the anterior position of the clasp [4]. At the same time, due to the large difference in the mechanical properties between plastics and metals, stress concentration at the connection causes problems, and RPDs are likely to fracture during long-term use [1]. Therefore, scholars are continuously exploring and seeking more aesthetically pleasing and durable materials, and polyether ether ketone (PEEK) has attracted attention as a biocompatible material with excellent performance [5].

As early as the 1990s, PEEK was used clinically as an implant material for applications such as artificial knee/hip joints and skull patches [6]. PEEK has been considered a high-quality dental implant material with good biocompatibility and mechanical properties [6,7], and it has a wide range of applications in the fabrication of fixed and removable dentures [8,9]. Most critically, PEEK, a high-performance polymer, can be combined with rapidly developing CAD-CAM technology to introduce new ideas for denture fabrication [7]. Zoidis et al. and Chen et al. reported that PEEK scaffolds produced less stress than metal scaffolds on abutment teeth and their periodontal ligament, and the authors recommended PEEK for denture restoration in patients with periodontal disease [7,10]. In recent years, some scholars have used digital technology to integrate the fabrication of PEEK obturators and removable partial denture frames and reported examples of clinical applications with ideal restorative results [9,11].

In recent years, with the widespread application of digital technology in the field of prosthodontics, some scholars have proposed the use of PEEK stents combined with acrylic resin dentures and heat-cured acrylic resin denture bases as an alternative to cobalt-chromium (Co–Cr) alloy stents. As a new material with good biocompatibility, mechanical properties and stress conduction, PEEK can not only improve the aesthetic effect of removable denture restoration but also eliminate patient discomfort, such as that due to allergic reactions to metals in traditional dentures [12].

Currently, the CAD-CAM PEEK RPD framework can be employed by directly milling the PEEK billet or by resin printing combined with thermal compression of PEEK using a lost wax technique to indirectly apply the material [13]. Studies have shown that the accuracies of the stents obtained by both techniques met the requirements for clinical application, but the accuracy was significantly higher for the stents obtained by direct fabrication techniques than those obtained by indirect techniques [14]. Arnold et al. found that the fit of RPD scaffolds fabricated by direct milling was superior to that of traditional cast metal scaffolds (43 ± 23 μm horizontal, 38 ± 21 μm vertical) [15]. Zoidis et al. and Lu et al. reported clinical cases of the use of RPD frameworks integrated with clasps made of PEEK where recovery of function and patient satisfaction received excellent evaluations [7,11].

The clasp, an important part of the RPD scaffold, has a great impact on both the aesthetics and function of the RPD. For clasps of different materials, retention and fit are key to their performance. In vitro, the retention performance of PEEK clasps is significantly inferior to that of metal clasps, while the deformation of PEEK clasps is not significantly different from that of metal clasps after cycles in simulation experiments [3,16]. Although the retention force of PEEK clasps is smaller than that of metal clasps, PEEK clasps meet clinical needs, and a combination of multiple clasps has been used to improve denture retention [17].

However, current scholars have different opinions on the feasibility of the clinical application of PEEK clasps, and there is a lack of systematic approach for experimental studies. Therefore, in this study, the feasibility of the clinical application of PEEK clasps was evaluated by designing PEEK clasps and Co–Cr clasps for different tooth positions and measuring their fit and retention force before and after 7200 cycles. The null hypotheses were that there would be no difference in fit between the PEEK clasps and the Co–Cr clasps, and the Co–Cr clasps would have better retention than the PEEK clasps.

## 2 Materials and methods

### 2.1 Design and fabrication of abutment teeth

Standard crowns (NISSIN, Kunshan, China) of the right mandibular first premolar (44) and first molar (46) were selected. The buccal surfaces were adjusted to form Class I leads where the undercut was far from the gap side, that is, the undercut was on the same side as the tip of the three-arm clasp. The undercut depth was greater than 0.5 mm. The adjacent surface was designed with a guide plane, which was parallel to the side edge of the square base, and the length was approximately two-thirds of the crown length.

After tooth preparation, model data of premolar 44 and molar 46 were obtained by scanning with a three-dimensional model dental scanner (3-Shape, Copenhagen, Denmark), and a square base of dimensions 10 mm × 10 mm x 10 mm was added.

The data were imported into a computer-controlled lathe for machining a five-axis linked denture (ARUM, Daejeon, Korea) and cutting a titanium metal block (Bego, Bremen, Germany) to obtain abutment teeth. Four metal abutments at position 44 and four at position 46 were fabricated as abutment models for subsequent clasp retention and fatigue cycles, respectively.

### 2.2 Design and fabrication of clasps

Circular three-arm clasps were designed on the abutments at positions 44 and 46 in 3 Shape dental design software (3-Shape, Copenhagen, Denmark) with 10 in each group, and the seating channel was flat with the side of the abutment base.

**Fig. 1:**
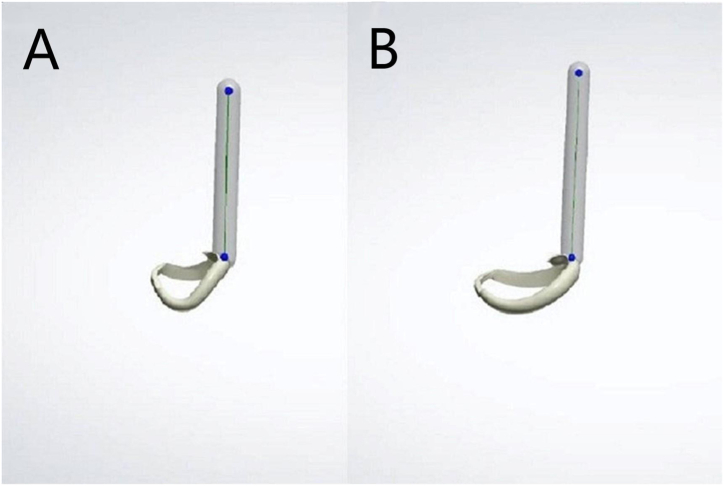
The addition of a cylindrical pull rod to the occlusal rests of the clasps (A-44; B-46).

The clasp design was undercut to 0.25 mm. A cylindrical pull rod with a diameter of 3 mm and length of 15 mm was placed parallel to the seating channel on the occlusal surface of the clasp occlusal rest to clamp the fixture to the universal dynamometer (Fig. 1). The thickest part of the Co–Cr clasp (body of the clasp) was approximately 1.0 mm, with a width of approximately 2.0 mm. The thickest part of the PEEK clasp was approximately 1.5 mm, with a width-to-thickness ratio of approximately 2:1. One-third of the end of the clasp was located in the undercut of the abutment teeth.

The cut PEEK clasps and cast Co–Cr clasps were fabricated according to the above design requirements. A total of N = 40 clasps were manufactured as follows.

**Fig. 2:**
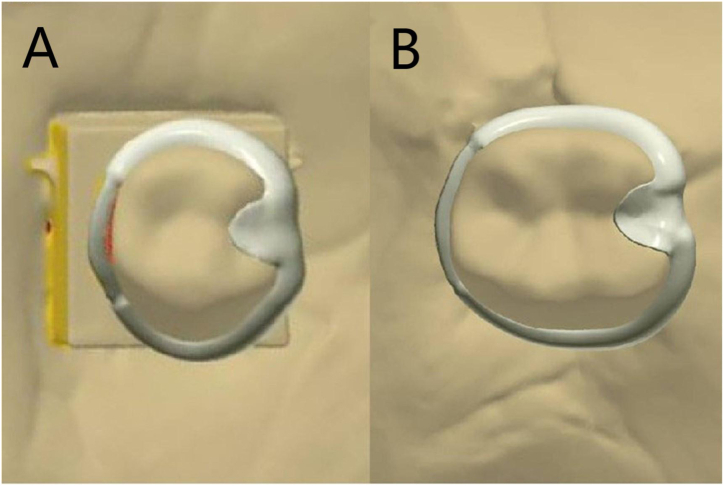
Design data for the wax molds of the cast clasps (A-44; B-46).

1. Fabrication of PEEK clasps: The designed data for two positions were imported into the computer-controlled machining lathe, which cut discs of polyether ether ketone resin (HuLiangShengWu, Shanghai, China) to obtain the PEEK clasps (n = 10 at each tooth position, total n_PEEK_ = 20).
2. Fabrication of Co–Cr clasps: A Co–Cr alloy block (Bego, Bremen, Germany) was cast to make the Co–Cr clasps using an intermediate frequency casting machine (HaiDeHaoTian, Tianjin, China) (n = 10 at each tooth position, total n_Co-Cr_ = 20). To ensure that the shape of the cast clasps was completely consistent with that of the PEEK clasps, the cast data were used to print a wax mold of the clasp by a resin printer. Next, the clasp was cast according to the traditional lost wax method to make the Co–Cr alloy clasp. The clasp was sandblasted with aluminum oxide particles with a diameter of 50 μm under a pressure of 500 kPa to ensure the consistency of the clasp shape. To prevent deformation when casting a clasp in the wax mold, a connecting rod was added to the clasp retention arm and the counter arm tip to form a closed loop (Fig. 2) before the Co–Cr clasp was cast.

### 2.3 Retention test

**Fig. 3:**
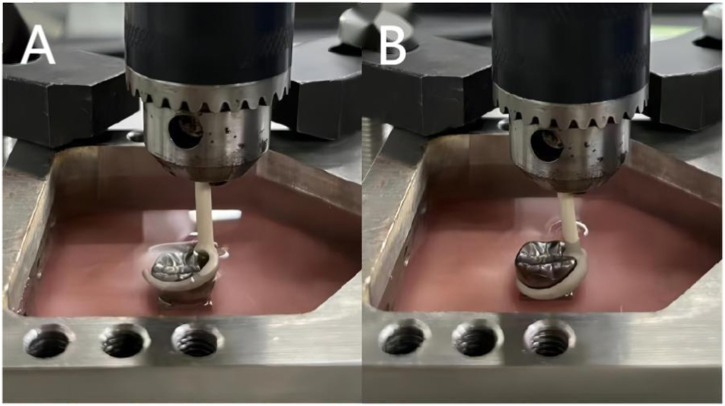
The retention force test for the clasp (PEEK clasp shown) (A-insetting; B-pull out).

**Fig. 4:**
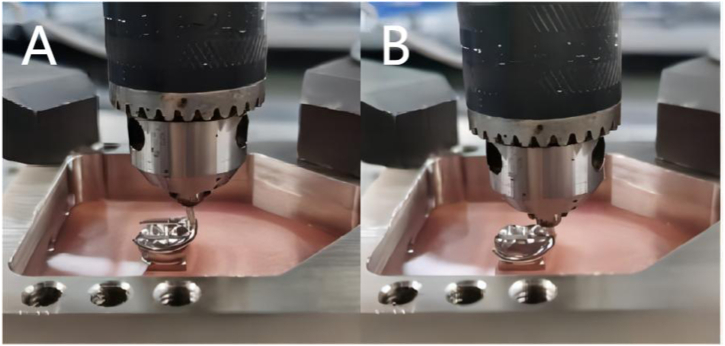
The retention force test for the clasp (Co–Cr clasp shown) (A-insetting; B-pull out).

The abutment teeth were fixed to the apparatus of a fatigue testing machine (DYNA-MESS, Aachen, Germany). The teeth were placed in artificial saliva that was composed of 0.4 g NaCl, 0.4 g KCl, 0.795 g CaCl_2_·2H_2_O, 0.78 g NaH_2_PO_4_·2H_2_O, 0.005 g Na_2_S·2H_2_O, 1 g CH_4_N_2_O and 1000 ml H_2_O. The clasp was pulled at a rate of 50 mm/min (Fig. 3, Fig. 4). The test cycle of 45 dislocations/min was repeated 7200 times to simulate dislocation for 5 years.

Each clasp (a total of N = 40) was cycled 7200 times under traction by the fatigue testing machine, and the maximum dislocation force was automatically recorded. The maximum dislocation force was recorded as the retention force, the average value of initial 5 dislocation forces of each fatigue cycle was recorded as the initial retention force of clasp, and the average value of the terminal 5 dislocation forces of each fatigue cycle was recorded as the terminal retention force of the clasp.

### 2.4 Fit test

The abutment teeth were fixed, and a light polyether silicone rubber body (DMG, Hamburg, Germany) was placed on the surfaces of the abutment teeth corresponding to the clasp. The clasp was pressed down to fit onto the abutment teeth, and a constant pressure of 9.8 N was applied to the clasp. After 5 min, the clasp was removed, and the thickness of the lightweight rubber at the clasp arm and support position were observed and measured using a stereomicroscope (Carl Zeiss, Jena, Germany).

The experiment was repeated after the clasp underwent 7200 fatigue cycles.

### 2.5 Statistical analysis

SPSS 23 statistical analysis software was used to analyze the above measurements. The independent-samples *t*-test was performed for different groups.

## 3 Results

### 3.1 Retention test

Before circulation, the measured retention forces of the clasps at position 44 were 4.61 ± 0.91 N (PEEK) and 47.50 ± 10.59 N (Co–Cr), and the retention forces at position 46 were 3.38 ± 0.49 N (PEEK) and 28.79 ± 10.99 N (Co–Cr). After circulation, the retention forces of the clasps at position 44 were 4.15 ± 0.91 N (PEEK) and 13.90 ± 6.59 N (Co–Cr), and the retention forces at position 46 were 2.93 ± 0.25 N (PEEK) and 11.56 ± 3.93 N (Co–Cr). (Table 1).

**Table 1:** Comparison of the retention force before and after cyclic testing at different tooth positions.

Group	Initial retention force		Terminal retention force	
	Mean (SD)	P	Mean (SD)	P
PEEK 44(n = 10)	4.61 (0.91)	0.002	4.15 (0.91)	0.002
PEEK 46(n = 10)	3.38 (0.49)		2.93 (0.25)	
Co–Cr 44(n = 10)	47.50 (10.59)	0.001	13.90 (6.59)	0.347
Co–Cr 46(n = 10)	28.79 (10.99)		11.56 (3.93)	

*The initial retention force of Co–Cr clasps was greater than that of PEEK clasps at the same tooth position.

*The retention forces of the clasps of the same material at the same tooth position decreased by different degrees after the clasps underwent cyclic testing, and the decrease in Co–Cr clasp retention was much greater than that in PEEK clasp retention.

*There was no significant difference in the retention force of the Co–Cr clasp at the two tooth positions after cyclic testing.

As shown in Table 1, the initial retention force was greater for Co–Cr clasps than PEEK clasps at the same tooth position, whereas the initial retention force was larger for clasps at position 44 than at position 46 for the same material. Additionally, the retention force of the clasps of the same material at the same tooth position decreased by varying degrees after the clasp underwent circulation, and the decrease in retention was much greater for Co–Cr clasps than PEEK clasps.

**Fig. 5:**
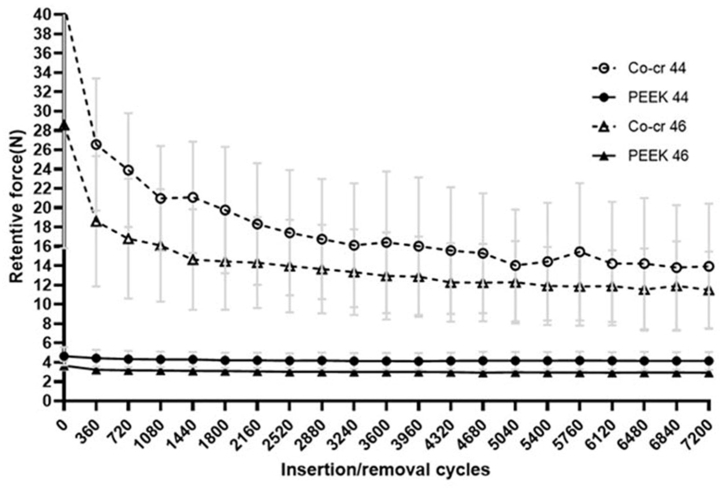
Change in forces required to remove clasps.

As shown in Fig. 5, the retention force at both tooth positions for Co–Cr clasps showed a large decrease after the fatigue cycle was performed approximately 360 times. Then, the retention force continued to decrease, but the amplitude was small and tended to be stable. In contrast, the retention force of the PEEK clasps remained in a relatively stable range throughout the cyclic fatigue testing.

#### 3.1.1 Statistical analysis

The independent-samples *t*-test was performed to assess the retention force after different numbers of fatigue cycles for clasps of the same material. The results are shown in Table 1.

For each material, the initial retention of the clasps at both tooth positions had P values < 0.05, indicating that the retention at position 44 was indeed greater than that at position 46 before cyclic fatigue testing. The P value of the terminal retention force of the PEEK clasps at the two tooth positions was <0.05, indicating that the retention force of the PEEK clasps was still greater at position 44 than position 46 after cyclic testing. The P value of the terminal retention force of the Co–Cr clasp at the two tooth positions was >0.05, indicating that there was no significant difference in the retention force of the Co–Cr clasp at the two tooth positions after testing.

The independent-samples *t*-test was performed on the reductions in clasp retention force for different materials at the same tooth position. The results are shown in Table 2.

**Table 2:** Comparison of the retention force reduction before and after cyclic testing at the same tooth position.

Group	Initial (N)	Terminal (N)	Reduction (N)	P
PEEK 44(n = 10)	4.61	4.15	0.46	＜0.001
Co–Cr 44(n = 10)	47.50	13.90	33.60	
PEEK 46(n = 10)	3.38	2.93	0.45	＜0.001
Co–Cr 46(n = 10)	28.79	11.56	17.23	

*There was a significant difference in the decrease in retention between the PEEK clasps and Co–Cr alloy clasps during fatigue cycles at the same tooth position.

The results (P < 0.05) indicated that there was a significant difference in the decrease in retention between the PEEK clasps and Co–Cr alloy clasps at the same tooth position during fatigue cycles. That is, the decrease in retention was less for the PEEK clasp than the Co–Cr alloy clasp.

### 3.2 Fit test

Table 3 shows that before circulation, the fit of each clasp at the reference points (clasp tip, clasp arm, and occlusal rest) was within 200 μm and that of the PEEK clasps was superior to that of the Co–Cr clasps at the same tooth positions before and after circulation. Moreover, the fit of the clasps of the same material at the same tooth position decreased after circulation. Before circulation, the sites of the tip and arm of clasps of the same material were more suitable at tooth position 44 than position 46, whereas the occlusal rest was more suitable at position 46 than position 44.

**Table 3:** Fit of clasps at reference points (clasp tip, clasp arm, and occlusal rest) for each tooth position before and after cyclic testing.

Group	Before circulation (μm)			After circulation (μm)		
	Clasp tip	Clasp arm	Occlusal rest	Clasp tip	Clasp arm	Occlusal rest
PEEK 46 (n = 10)	66.25 ± 29.43	41.70 ± 32.41	110.54 ± 75.38	64.05 ± 29.50	64.47 ± 23.74	109.49 ± 50.03
Co–Cr 46 (n = 10)	70.63 ± 22.37	54.23 ± 28.51	170.29 ± 83.03	104.51 ± 47.91	80.71 ± 46.55	182.59 ± 97.89
PEEK 44 (n = 10)	70.84 ± 29.40	43.24 ± 32.50	79.93 ± 38.87	79.07 ± 25.79	66.28 ± 21.86	110.52 ± 43.44
Co–Cr 44 (n = 10)	71.82 ± 35.34	65.92 ± 75.43	99.28 ± 43.82	100.28 ± 41.98	84.61 ± 37.35	147.62 ± 113.94

#### 3.2.1 Statistical analysis

Independent-samples t tests were performed for the fit of different materials at the same tooth position after cyclic fatigue testing of the clasps. The results are shown in Table 4, Table 5.

**Table 4:** Comparison of the fit of clasps with different materials in the same tooth position before cyclic testing.

Group	Before circulation (μm)					
	Clasp tip	P	Clasp arm	P	Occlusal rest	P
PEEK 46 (n = 10)	66.25 ± 29.43	0.712	41.70 ± 32.41	0.371	110.54 ± 75.38	0.109
Co–Cr 46 (n = 10)	70.63 ± 22.37		54.23 ± 28.51		170.29 ± 83.03	
PEEK 44 (n = 10)	70.84 ± 29.40	0.947	43.24 ± 32.50	0.394	79.93 ± 38.87	0.335
Co–Cr 44 (n = 10)	71.82 ± 35.34		65.92 ± 75.43		99.28 ± 43.82	

*Before testing, there was no statistically significant difference in the fits of the PEEK clasp and Co–Cr alloy clasp at the same tooth position.

**Table 5:** Comparison of the fit of clasps with different materials in the same tooth position after testing.

Group	After circulation (μm)					
	Clasp tip	P	Clasp arm	P	Occlusal rest	P
PEEK 46 (n = 10)	64.05 ± 29.50	0.035	64.47 ± 23.74	0.339	109.49 ± 50.03	0.055
Co–Cr 46 (n = 10)	104.51 ± 47.91		80.71 ± 46.55		182.59 ± 97.89	
PEEK 44 (n = 10)	79.07 ± 25.79	0.194	66.28 ± 21.86	0.197	110.52 ± 43.44	0.349
Co–Cr 44 (n = 10)	100.28 ± 41.98		84.61 ± 37.35		147.62 ± 113.94	

*After testing, the fit of the PEEK clasp tip at tooth position 46 was better than that of the Co–Cr alloy clasp.

Before circulation, the P values were >0.05 at each reference point; that is, there was no statistically significant difference in the fits of the PEEK clasps and Co–Cr alloy clasps at the same tooth position (Table 4).

After circulation, the P value of the clasp tips at tooth position 46 was <0.05, and the P values at the other reference points were >0.05. That is, the fit of the PEEK clasp tip at tooth position 46 was better than that of the Co–Cr alloy clasp (Table 5).

The independent-samples *t*-test was performed for the fit at different tooth positions for clasps of the same material after cyclic fatigue testing. The results are shown in Table 6, Table 7.

**Table 6:** Comparison of the fit of clasps of different tooth positions with the same material before testing.

Group	Before circulation (μm)					
	Clasp tip	P	Clasp arm	P	Occlusal rest	P
PEEK 46 (n = 10)	66.25 ± 29.43	0.731	41.70 ± 32.41	0.917	110.54 ± 75.38	0.269
PEEK 44 (n = 10)	70.84 ± 29.40		43.24 ± 32.50		79.93 ± 38.87	
Co–Cr 46 (n = 10)	70.63 ± 22.37	0.929	54.23 ± 28.51	0.652	170.29 ± 83.03	0.030
Co–Cr 44 (n = 10)	71.82 ± 35.34		65.92 ± 75.43		99.28 ± 43.82	

*Before testing, there was no statistically significant difference in the fit of the PEEK clasp at different tooth positions.

*There was a statistically significant difference in the fit of the Co–Cr clasp at the occlusal rest points at tooth positions 44 and 46.

**Table 7:** Comparison of the fit of clasps of different tooth positions with the same material after testing.

Group	After circulation (μm)					
	Clasp tip	P	Clasp arm	P	Occlusal rest	P
PEEK 46 (n = 10)	64.05 ± 29.50	0.241	64.47 ± 23.74	0.861	109.49 ± 50.03	0.961
PEEK 44 (n = 10)	79.07 ± 25.79		66.28 ± 21.86		110.52 ± 43.44	
Co–Cr 46 (n = 10)	104.51 ± 47.91	0.836	80.71 ± 46.55	0.839	182.59 ± 97.89	0.471
Co–Cr 44 (n = 10)	100.28 ± 41.98		84.61 ± 37.35		147.62 ± 113.94	

*After testing, there was no statistically significant difference in the fits of the clasps of the same material at different tooth positions.

Before cyclic testing, the P values of the PEEK clasp at each reference point of tooth positions 44 and 46 were >0.05. That is, there was no statistically significant difference in the fit of the PEEK clasp at different tooth positions. In contrast, the P value of the Co–Cr clasps at the support part was <0.05, and the P values at the other reference points were >0.05, indicating a statistically significant difference in the fit of the Co–Cr clasps at the occlusal rest points at tooth positions 44 and 46 (Table 6).

After circulation, the reference points of the PEEK clasps and Co–Cr clasps at tooth positions 44 and 46 were P > 0.05. That is, there was no statistically significant difference in the fits of the clasps of the same material at different tooth positions (Table 7).

## 4 Discussion

In this experiment, we designed 0.25 mm undercuts on teeth at two positions and simulated cyclic fatigue for 5 years of use. The fatigue test was based on removing dentures 4 times per day and 30 days per month, which corresponds to a total of 7200 removals over 5 years. Our goal was to further verify the feasibility of the clinical application of PEEK clasps through systematic tests [17]. The results of the retention and fit test showed that the retention force was larger for Co–Cr clasps than PEEK clasps before and after cyclic fatigue tests, whereas there was no difference in the fits of the clasps for the two materials at the same tooth position before cyclic testing. Therefore, the null hypothesis that there would be no difference in fit between the PEEK clasps and the Co–Cr clasps, whereas the Co–Cr clasps would have better retention than the PEEK clasps, was accepted.

According to the data from this experimental study, the retention force was significantly greater for Co–Cr clasps than PEEK clasps before and after cyclic testing, but the retention force decreased less for the PEEK clasps than the Co–Cr clasp during 7200 cycles. This may have been related to the lower elastic modulus of PEEK, which has better flexibility than Co–Cr. The retention force of the Co–Cr clasps decreased significantly after circulation for approximately 360 cycles, which showed that there would be a significant decrease in retention performance after 90 days of use. This was not the case with the PEEK clasps. There was no statistically significant difference in the fits of the PEEK and Co–Cr clasps at the same tooth position before fatigue cycles. Then the Co–Cr clasp tip at tooth position 46 underwent greater deformation than the PEEK clasp after the cycles of testing. Marie et al. also showed that although the Co–Cr clasp still had a large retention force after 15,000 seating-dislocation cycles, more significant deformation occurred [18]. This was corroborated by the experimental results of the changes in clasp retention performance. In the study of conventional cast RPDs by Dunham et al. the fit of the occlusal rest was 193 ± 203 μm, and the fit at each site of the clasp in this experiment was within that range [19]. In addition, the consistency in the fits of the clasps for the two materials before circulation could have indicated that the PEEK clasp was suitable to meet clinical needs. The initial retention force of the PEEK clasp was 3.38–4.61 N, and the terminal retention force was 2.93–4.15 N, which could meet clinical needs. Some scholars have reported that the total retention force of a pair of dentures with 2∼4 clasps in the general design could meet clinical needs when it reached approximately 7.8 Ñ14.7 N [17]. Thus, it could be concluded that the retention ability of the PEEK clasps could meet the requirements for clinical applications through reasonable denture design.

Scholars still hold different opinions about the feasibility of the clinical application of PEEK clasps. PEEK clasps have been recognized for providing good cosmetic results, but they have significantly lower retention X than conventional metal clasps. It is generally believed that the retention force of a single clasp correlates with the length of the clasp arm, the contact area with the abutment tooth and the depth of entry into the undercut. Some scholars have proposed that the lack of a small retention force of a PEEK clasp can be compensated for by increasing the depth of the inverted concave, but some studies have noted that when the inverted concave is increased, the removal of the clasp produces greater stress, causing adverse effects on the repair effect [16].

Tannous et al. stated that in vitro studies showed that designing resin clasps for 0.5 mm undercuts could improve not only retention but also aesthetics [3]. In this experiment, only 0.25 mm undercuts were designed due to the limitation of abutment tooth morphology. However, in combination with reports in the literature, it can be assumed that the retention force of the PEEK clasp may be improved when designing a 0.5 mm undercut depth. Additionally, more experiments are needed to confirm the possibility that excessive stress occurs when the clasp enters deeper recesses.

There is also a correlation between the shape of the clasp and the retention effect. Peng et al. designed 3D models of clasps with different thicknesses, widths and clasp arm tapers and simulated the removal and wearing cycles of PEEK clasps and Co–Cr clasps for 10 years in combination with finite element analysis (FEA). The results showed that the retention force of PEEK clasps was significantly correlated with their cross-sectional area, while the same thickness of X had a greater effect on the retention force of clasps when the cross-sectional area was measured [20]. Their results showed that a PEEK clasp width 3.0 mm, thickness 1.88 mm and taper 1.0 met the minimum clinical requirement (1.6 N) when the undercut was 0.25 mm. In this experiment, a PEEK clasp with 3.0 mm width and 1.5 mm thickness under 0.25 mm inverted recess was designed, and the measured retention force could meet clinical needs. However, there is no more detailed classification method for the data of clasp tapers, etc. In addition, it is necessary to include considerations of whether the width and thickness of the clasp increase will affect the comfort of the patient during use in clinical design.

Limitations of this experiment included the use of a fatigue testing machine to simulate the 5-year removal procedure. The set force direction of the machine was consistent with the seating channel. However, the actual process during clinical use would not ensure that the force direction of the finger would be consistent with the seating channel for each removal and insertion. In addition, there were differences in hardness and wear resistance between metal abutments and natural teeth, which resulted in the inability to completely simulate the intraoral situation. Therefore, more cycles are required to simulate longer-term use of PEEK clasps and to further evaluate their feasibility for clinical use.

In conclusion, under the limited conditions of this experiment, the PEEK clasps met the requirements for clinical applications. Compared with the Co–Cr clasps, the PEEK clasps underwent less fatigue deformation.

## Author contribution statement

Su Wu: Performed the experiments; Analyzed and interpreted the data; Contributed reagents, materials, analysis tools or data; Wrote the paper. Chao Qian: Analyzed and interpreted the data. Ting Jiao: Conceived and designed the experiments. Jian Sun: Tutor, Conceived and designed the experiments; Contributed reagents, materials, analysis tools or data.

## Data availability statement

Data will be made available on request.

## Declaration of competing interest

The authors declare that they have no known competing financial interests or personal relationships that could have appeared to influence the work reported in this paper.
